# Prevalence and Risk Factors of Nonmedical Prescription Opioid Use Among Transgender Girls and Young Women

**DOI:** 10.1001/jamanetworkopen.2020.1015

**Published:** 2020-03-16

**Authors:** Arjee J. Restar, Harry Jin, Adedotun Ogunbajo, William C. Goedel, Gregorio Millett, Jennifer Sherwood, Lisa Kuhns, Sari L. Reisner, Robert Garofalo, Matthew J. Mimiaga

**Affiliations:** 1 Department of Behavioral and Social Sciences, Brown University School of Public Health, Providence, Rhode Island; 2 Center for Health Equity Research, Brown University, Providence, Rhode Island; 3 amfAR, The Foundation for AIDS Research, New York, New York; 4 Department of Epidemiology, Brown University School of Public Health, Providence, Rhode Island; 5 Division of Adolescent Medicine, Ann and Robert H. Lurie Children’s Hospital, Chicago, Illinois; 6 Feinberg School of Medicine, Department of Pediatrics, Northwestern University, Chicago, Illinois; 7 Boston Children’s Hospital, Division of General Pediatrics, Harvard Medical School, Boston, Massachusetts; 8 Department of Epidemiology, Harvard T.H. Chan School of Public Health, Boston, Massachusetts; 9 The Fenway Institute, Fenway Health, Boston, Massachusetts

## Abstract

**Importance:**

Nonmedical prescription opioid use is a pressing public health issue in the United States. Transgender youth, including adolescent girls and young women who were assigned male at birth and currently identify as women, female, transgender women, or another diverse gender identity along the transfeminine gender spectrum, are more likely than their cisgender peers to report illicit substance use and meet diagnostic criteria for substance use disorders. However, relatively little is known about the experiences of these populations in the current era of opioid addiction and misuse.

**Objective:**

To report the prevalence of and risk factors associated with lifetime nonmedical prescription opioid use in a high-risk community sample of transgender adolescent girls and young women who are sexually active.

**Design, Setting, and Participants:**

This cross-sectional study used 2012 to 2015 baseline data from Project LifeSkills, a randomized clinical trial of a behavioral intervention to reduce the risk of HIV acquisition and transmission among a diverse sample of transgender adolescent girls and young women recruited from Boston, Massachusetts, and Chicago, Illinois. A total of 297 transgender girls and women aged 16 to 29 years who were sexually active were included in this analysis. Data were analyzed from June 2019 to August 2019.

**Exposures:**

Transgender woman identification.

**Main Outcomes and Measures:**

Self-reported lifetime nonmedical prescription opioid use.

**Results:**

Among 297 transgender adolescent girls and young women (mean [SD] age, 23.4 [3.5] years), 145 (48.8%) identified as non-Hispanic/Latinx black, 76 (25.6%) identified as non-Hispanic/Latinx white, 37 (12.5%) identified as Hispanic/Latinx, 7 (2.4%) identified as non-Hispanic/Latinx Asian, and 32 (10.8%) identified as multiracial or other race/ethnicity. Thirty-five participants (11.8%) reported lifetime nonmedical prescription opioid use. Young transgender women who smoked cigarettes monthly or less (adjusted odds ratio, 3.92; 95% CI, 1.10-13.89) and who smoked daily (adjusted odds ratio, 5.69; 95% CI, 1.87-17.33) had greater odds of nonmedical prescription opioid use compared with those who did not smoke. Additionally, participants who identified as a sexual orientation other than heterosexual, gay, lesbian, or bisexual had significantly greater odds of lifetime nonmedical prescription opioid use compared with those who identified as heterosexual (adjusted odds ratio, 3.69; 95% CI, 1.07-12.72).

**Conclusions and Relevance:**

These findings suggest that transgender adolescent girls and young women have similar prevalence of lifetime nonmedical prescription opioid use compared with the US general population prevalence of 12.5%. These findings may serve as a call-to-action for public health surveillance studies and evidence-based interventions to be comprehensively tailored to examine and respond to specific trends of substance use, particularly opioid use disorder, among transgender populations.

## Key Points

**Question:**

What are the prevalence of and risk factors associated with lifetime nonmedical prescription opioid use among transgender adolescent girls and young women who are sexually active?

**Findings:**

In this cross-sectional study of 297 transgender girls and young women, 35 participants (11.8%) reported lifetime nonmedical prescription opioid use. Risk factors for opioid use included smoking cigarettes and identifying as a sexual orientation other than gay, lesbian, or bisexual.

**Meaning:**

These findings suggest that evidence-based services for substance use, particularly those specifically aimed at treating opioid use disorder, can and should be tailored comprehensively to transgender populations, particularly transgender girls and young women.

## Teaser

This cross-sectional study examines the prevalence and risk factors associated with nonmedical prescription opioid use among transgender women and girls aged 16 to 29 years.

## Introduction

Deaths attributable to accidental drug overdose, particularly those involving opioids, have reached epidemic levels in the United States, with more than 350 000 documented opioid overdose deaths between 2000 and 2015.^1,2^ The opioid overdose epidemic has been described as a *triple-wave epidemic*, with a first wave of opioid overdoses associated with prescription opioids beginning in the late 1990s and early 2000s, a second wave of opioid overdoses associated with heroin beginning the middle of the 2000s, and a third wave of opioid overdoses associated with fentanyl, fentanyl analogues, and other synthetic opioids beginning in the early 2010s.^3^ There is significant regional variation in the burden of opioid addiction and misuse, with the highest age-adjusted death rates due to opioid overdose observed in New England and the Midwest.^1,2^

Nonmedical prescription opioid use is a pressing public health issue in the United States.^4^ Given the dramatic increases in availability of prescription opioids in the United States during the past 20 years, results from a 2015 national study^4^ suggest that the most recent generations of young adults have experienced earlier initiation of nonmedical prescription opioid use and have been at higher risk for transitioning to heroin use than previous generations. As a result, young adults engaging in nonmedical prescription opioid use are at increasingly high risk of adverse outcomes associated with drug use, such as increasing rates of unintentional overdose and acquisition of bloodborne infections.^5,6,7^ As of 2017, 1 in 8 individuals aged 18 to 35 years report nonmedical prescription opioid use in their lifetime.^8^ Thus, documenting the prevalence of nonmedical prescription opioid use among young adults at increased risk and identifying factors associated with use may help inform programs and policies designed to reduce opioid use and drug-related harms in this population.

Transgender individuals are more likely to report illicit substance use and meet diagnostic criteria for substance use disorders compared with their cisgender peers,^9,10,11,12,13^ but relatively little is known about the experiences of these populations in the current era of opioid addiction and misuse. A recent analysis of the National Survey of Drug Use and Health found that gay, lesbian, and bisexual individuals were more likely than their heterosexual counterparts to report nonmedical prescription opioid use in their lifetimes (20.2% vs 10.2%), in the past 12 months (9.0% vs 4.0%), and in the past 30 days (2.7% vs 1.2%).^14^ However, to our knowledge, no national probability-based surveys of substance use and related outcomes currently include measures of both nonmedical prescription opioid use and gender identity, precluding any ability to understand the burden of opioid misuse in gender minority populations relative to cisgender groups. To begin to fill in this gap in the literature and explore this behavioral outcome in an understudied community, we estimated the prevalence of nonmedical prescription opioid use among a convenience sample of young transgender women in 2 urban centers heavily affected by opioid addiction and misuse and identify factors associated with this use.

## Methods

This cross-sectional study leverages baseline data from Project LifeSkills,^15^ a randomized clinical trial of a behavioral intervention to reduce the risk of HIV acquisition and transmission among transgender adolescent girls and young women. The study protocols and related methods have been described in detail elsewhere.^16^ In brief, participants were recruited between 2012 and 2015 in Boston, Massachusetts, and Chicago, Illinois, using multiple convenience sampling methods. Individuals were eligible for participation in the study if they were between ages 16 to 29 years, assigned male at birth and now identify as transgender along the transfeminine spectrum (eg, as a woman, female, transgender woman), spoke English, and self-reported engaging in condomless vaginal or anal intercourse in the past 12 months. All participants provided written informed consent. The institutional review board at both study sites approved all study protocols. This study is reported following the Strengthening the Reporting of Observational Studies in Epidemiology (STROBE) reporting guideline.

Enrolled participants completed a baseline questionnaire that collected information on various domains, including sociodemographic characteristics, social marginalization, and psychosocial factors (eg, alcohol and illicit drug use). Participants were screened for HIV infection and other bacterial sexually transmitted infections. To assess lifetime nonmedical prescription opioid use, we asked participants, “Have you ever used pain killer (e.g., Percocet, Percodan, OxyContin, oxycodone, codeine) without a doctor’s prescription?”^17^ Three participants did not respond to this question and were removed from the analytic data set. Participants who responded yes to this question were categorizing as having ever engaged in nonmedical prescription opioid use. The baseline data of the remaining participants were included in this analysis.

In line with our primary aim, we explored variables associated with nonmedical prescription opioid use, including demographic characteristics (eg, age, race/ethnicity, educational attainment), experiences of social marginalization (eg, criminal justice involvement, experiences of homelessness, engagement in sex work), psychosocial processes (eg, suicidal ideation, symptoms of depression and anxiety, use of alcohol and other substances), and indicators of physical and mental health care access (eg, access to a primary care practitioner, current insurance coverage, avoidance of health care services owing to discrimination). Sexual orientation variables were based on how participants identified themselves.

### Statistical Analysis

The associations of lifetime nonmedical opioid use with participant characteristics were compared using *t* tests for continuous variables and χ^2^ tests for categorical variables. Factors associated with lifetime nonmedical opioid use were identified using logistic regression procedures. In the final adjusted model, the inclusion criteria for including variables were all demographic characteristics as well as all other variables found to be associated with lifetime nonmedical prescription opioid use in unadjusted analyses. All analyses were conducted in SAS statistical software version 9.4 (SAS Institute). *P* values were 2-tailed, and statistical significance was set at .05. Data were analyzed from June 2019 to August 2019.

## Results

### Sample Characteristics

Among 297 transgender adolescent girls and young women (mean [SD] age, 23.4 [3.5] years), 145 (48.8%) identified as non-Hispanic/Latinx black, 76 (25.6%) identified as non-Hispanic/Latinx white, 37 (12.5%) identified as Hispanic/Latinx, 7 (2.4%) identified as non-Hispanic/Latinx Asian, and 32 (10.8%) identified as multiracial or other race/ethnicity (Table 1). A total of 35 participants (11.8%) reported nonmedical prescription opioid use in their lifetimes.

**Table 1.** Characteristics of a Sample of Young Transgender Girls and Women Stratified by Lifetime Nonmedical Prescription Opioid Use

Characteristic	No. (%)			*P* Value
	Total (N = 297)	Lifetime Nonmedical Prescription Opioid Use		
		No (n = 262)	Yes (n = 35)	
Study site				
Boston, Massachusetts	144 (48.5)	121 (46.2)	23 (65.7)	.03
Chicago, Illinois	153 (51.5)	141 (53.8)	12 (34.3)	
Age, mean (SD), y	23.4 (3.5)	23.2 (3.5)	24.8 (3.2)	.009
Sexual orientation				
Heterosexual	116 (39.1)	102 (38.9)	14 (40.0)	.049
Gay or homosexual	79 (26.6)	73 (27.9)	6 (17.1)	
Lesbian	16 (5.4)	15 (5.7)	1 (2.9)	
Bisexual	58 (19.5)	52 (19.9)	6 (17.1)	
Other	28 (9.4)	20 (7.6)	8 (22.9)	
Race/ethnicity				
Non-Hispanic/Latinx				.16
White	76 (25.6)	63 (24.1)	13 (37.1)	
Black	145 (48.8)	135 (51.5)	10 (28.6)	
Asian	7 (2.4)	6 (2.3)	1 (2.9)	
Hispanic/Latinx	37 (12.5)	31 (11.8)	6 (17.1)	
Multiracial or other	32 (10.8)	27 (10.3)	5 (14.3)	
National origin				
United States	284 (95.6)	251 (95.8)	33 (94.3)	.68
Outside the United States	13 (4.4)	11 (4.2)	2 (5.7)	
Educational attainment				
≤High school degree	174 (58.6)	157 (59.9)	17 (48.6)	.20
≥Some college	123 (41.4)	105 (40.1)	18 (51.4)	
Household income, $				
<10 000	136 (45.8)	119 (45.4)	17 (48.6)	.63
≥10 000	91 (30.6)	79 (30.2)	12 (34.3)	
Don’t know or not sure	70 (23.6)	64 (24.4)	6 (17.1)	
Ever experienced arrest or incarceration				
Yes	163 (54.9)	137 (52.3)	26 (74.3)	.01
No	134 (45.1)	125 (47.7)	9 (25.7)	
Recent experience of homelessness				
Yes	71 (23.9)	61 (23.3)	10 (28.6)	.49
No	226 (76.1)	201 (76.7)	25 (71.4)	
Recent engagement in sex work				
Yes	104 (35.0)	89 (34.0)	15 (42.9)	.30
No	193 (65.0)	173 (66.0)	20 (57.1)	
Recent experience of major depressive episode				
Yes	42 (14.1)	34 (13.0)	8 (22.9)	.12
No	255 (85.9)	228 (87.0)	27 (77.1)	
Recent symptoms				
Generalized anxiety disorder				
Yes	112 (37.7)	96 (36.8)	16 (45.7)	.55
No	184 (62.0)	165 (63.2)	19 (54.3)	
Posttraumatic stress disorder				
Yes	25 (8.5)	19 (7.3)	6 (17.1)	.05
No	269 (91.5)	240 (92.7)	29 (82.9)	
Alcohol use disorder				
Yes	32 (10.8)	24 (9.2)	8 (22.9)	.01
No	265 (89.2)	238 (90.8)	27 (77.1)	
Substance use disorder				
Yes	43 (14.6)	34 (13.1)	9 (25.7)	.046
No	252 (85.4)	228 (86.9)	26 (74.3)	
Mean alcoholic drinks consumed when drinking, past 4 mo, No.				
Nondrinker	108 (36.4)	100 (38.2)	8 (22.9)	.27
≤1	34 (11.5)	29 (11.1)	5 (14.3)	
2-3	97 (32.7)	85 (32.4)	12 (34.3)	
>4	58 (19.5)	48 (18.3)	10 (28.6)	
Current cigarette smoking frequency				
None	147 (49.5)	140 (53.4)	7 (20.0)	.002
≤Monthly	45 (15.2)	38 (14.5)	7 (20.0)	
Weekly	25 (8.4)	21 (8.0)	4 (11.4)	
Daily	80 (26.9)	63 (24.1)	17 (48.6)	
Current access to primary care practitioner				
Yes	212 (71.4)	185 (70.6)	27 (77.1)	.42
No	85 (28.6)	77 (29.4)	8 (22.9)	
Current health insurance coverage				
Yes	221 (74.4)	194 (74.1)	27 (77.1)	.69
No	76 (25.6)	68 (26.0)	8 (22.9)	
Avoidance of physical health services				
Owing to discrimination				
Yes	58 (19.5)	49 (18.7)	9 (25.7)	.33
No	239 (80.5)	213 (81.3)	26 (74.3)	
Owing to cost				
Yes	80 (26.9)	70 (26.7)	10 (28.6)	.82
No	217 (73.1)	192 (72.3)	25 (71.4)	
Current access to mental health care practitioner				
Yes	111 (37.4)	98 (37.4)	13 (37.1)	.98
No	186 (62.6)	164 (62.6)	22 (62.9)	
Current health insurance coverage for mental health				
Yes	192 (64.7)	167 (63.7)	25 (71.4)	.37
No	105 (35.4)	95 (36.3)	10 (28.6)	
Avoidance of mental health services				
Owing to discrimination				
Yes	29 (9.8)	21 (8.0)	8 (22.9)	.006
No	268 (90.2)	241 (92.0)	27 (77.1)	
Owing to cost				
Yes	67 (22.6)	56 (21.4)	11 (31.4)	.18
No	230 (77.4)	206 (78.6)	24 (68.6)	

### Factors Associated With Nonmedical Prescription Opioid Use

The results of the unadjusted and adjusted logistic regressions are presented in Table 2. In the unadjusted logistic regression models, participants recruited in Chicago, Illinois, had lower odds of nonmedical prescription opioid use compared with participants who were recruited in Boston, Massachusetts (odds ratio [OR], 0.45; 95% CI, 0.21-0.94). Increasing age was associated with greater odds of nonmedical prescription opioid use (OR per 1-year increase, 1.15; 95% CI, 1.03-1.27). Compared with participants who identified as heterosexual, those who identified as a sexual orientation other than heterosexual, gay, lesbian, or bisexual had greater odds of nonmedical prescription opioid use (OR, 2.92; 95% CI, 1.08-7.86). Black participants had lower odds of nonmedical prescription opioid use compared with white participants (OR, 0.36; 95% CI, 0.15-0.86). Participants who ever experienced arrest or incarceration (OR, 2.64; 95% CI, 1.19-5.84), recently experienced symptoms of alcohol use disorder (OR, 2.94; 95% CI, 1.20-7.18), recently experienced symptoms of substance use disorder (OR, 2.32; 95% CI, 1.00-5.37), or reported having avoided accessing mental health services owing to discrimination (OR, 3.40; 95% CI, 1.37-8.42) had greater odds of nonmedical prescription opioid use compared with those who did not. Participants who smoked cigarettes monthly or less (OR, 3.68; 95% CI, 1.22-11.15), weekly (OR, 3.81; 95% CI, 1.03-14.14), or daily (OR, 5.40; 95% CI, 2.13-13.67) had greater odds of nonmedical prescription opioid use compared with those who did not smoke cigarettes.

**Table 2.** Odds of Lifetime Nonmedical Prescription Opioid Use Among Young Transgender Girls and Women

Characteristic	Odds Ratio (95% CI)	
	Unadjusted	Adjusted
Study site		
Boston, Massachusetts	1 [Reference]	1 [Reference]
Chicago, Illinois	0.45 (0.21-0.94)	0.66 (0.24-1.77)
Age, per 1-y increase	1.15 (1.03-1.27)	1.10 (0.96-1.26)
Sexual orientation		
Heterosexual	1 [Reference]	1 [Reference]
Gay or homosexual	0.60 (0.22-1.63)	0.84 (0.27-2.68)
Lesbian	0.49 (0.06-3.97)	0.60 (0.06-6.43)
Bisexual	0.84 (0.31-2.32)	0.89 (0.27-2.91)
Other	2.92 (1.08-7.86)	3.69 (1.07-12.72)
Race/ethnicity		
Non-Hispanic/Latinx		
White	1 [Reference]	1 [Reference]
Black	0.36 (0.15-0.86)	0.50 (0.16-1.63)
Asian	0.81 (0.09-7.29)	1.59 (0.07-35.30)
Hispanic/Latinx	0.94 (0.33-2.70)	1.96 (0.52-7.31)
Multiracial or other	0.90 (0.29-2.77)	0.83 (0.20-3.44)
National origin		
United States	1 [Reference]	1 [Reference]
Outside the United States	1.38 (0.29-6.52)	0.73 (0.07-8.22)
Educational attainment		
≤High school degree	1 [Reference]	1 [Reference]
≥Some college	1.58 (0.78-3.21)	1.18 (0.47-2.98)
Household income, $		
<10 000	1 [Reference]	1 [Reference]
≥10 000	1.06 (0.48-2.35)	1.11 (0.44-2.83)
Don’t know or not sure	0.66 (0.25-1.75)	0.82 (0.27-2.54)
Ever experienced arrest or incarceration^a^	2.64 (1.19-5.84)	2.36 (0.82-6.81)
Recent symptoms of alcohol use disorder^a^	2.94 (1.20-7.18)	1.29 (0.41-4.09)
Recent symptoms of substance use disorder^a^	2.32 (1.00-5.37)	1.54 (0.50-4.74)
Current cigarette smoking frequency		
None	1 [Reference]	1 [Reference]
≤Monthly	3.68 (1.22-11.15)	3.92 (1.10-13.89)
Weekly	3.81 (1.03-14.14)	3.76 (0.80-17.66)
Daily	5.40 (2.13-13.67)	5.69 (1.87-17.33)
Avoidance of mental health services owing to discrimination^a^	3.40 (1.37-8.42)	2.73 (0.89-8.40)

^a^ Calculated using responding no for the characteristic as the reference.

In the adjusted logistic regression, participants who identified as a sexual orientation other than heterosexual, gay, lesbian, or bisexual had significantly greater odds of nonmedical prescription opioid use compared with participants who identified as heterosexual (adjusted OR, 3.69; 95% CI, 1.07-12.72). Additionally, participants who reported smoking cigarettes monthly or less (adjusted OR, 3.92; 95% CI, 1.10-13.89) and participants who smoked daily (adjusted OR, 5.69; 95% CI, 1.87-17.33) had greater odds of nonmedical prescription opioid use compared with participants who did not smoke cigarettes.

## Discussion

To our knowledge, this is the first empirical study that examined self-reported lifetime nonmedical prescription opioid use among a community-recruited sample of transgender adolescent girls and young women in 2 cosmopolitan cities in the United States. Our findings provide insights for this group’s nonmedical use of prescription opioids at a time and context when the United States is experiencing an escalating opioid epidemic.^18^ Results of this study suggest similar lifetime nonmedical prescription opioid use among this group compared with the national prevalence.^8^ Specifically, transgender adolescent girls and young women who are current cigarette smokers and transgender adolescent girls and young women who identified as a sexual orientation other than heterosexual, gay, lesbian, or bisexual had significantly higher odds of lifetime nonmedical prescription opioid use than their counterparts.

We found that 11.8% of transgender adolescent girls and young women in our sample reported lifetime nonmedical prescription opioid use, compared with a national prevalence of 12.5% lifetime nonmedical prescription opioid use among their cisgender counterparts.^8^ These similar prevalence estimates indicate a need for inclusive and culturally sensitive substance use intervention solutions and approaches among transgender populations, particularly transgender adolescent girls and young women. A 2017 systematic review of problematic substance use among transgender populations^19^ found that only 2 studies have designed substance use–related interventions for this group, and no studies aimed to reduce nonmedical prescription opioid use. The prevalence of lifetime nonmedical prescription opioid use among transgender adolescent girls and young women in this sample highlights the need for evidence-based substance use–related interventions for this group. Furthermore, it has been suggested that rates of substance use among sexual and gender minority populations can vary by subgroup and the type of drug used.^9,14^ In addition to addressing nonmedical prescription opioid use, there is a need to understand and characterize trends of heroin and fentanyl use among transgender populations, given how these 2 substances have also been associated with opioid-related overdoses and deaths in the United States.^18,20^ As such, given that this study specifically only asked about lifetime nonmedical prescription opioid use between 2012 to 2015, there is a need for current or future surveillance studies to examine patterns of current or recent lifetime nonmedical prescription opioid use, types of opioids used, and polysubstance use among this group to inform evidence-based substance use–related interventions for transgender adolescent girls and young women. Moreover, given that the history of opioid use among this sample was similar to the current national prevalence, it is important for national surveillance systems to begin collecting data specific to gender identity to understand trends for this group’s opioid-related behavior over time.

There are several possible explanations for why girls and women in our sample who reported having sexual orientation other than heterosexual, gay, lesbian, or bisexual had higher nonmedical prescription opioid use. The first reason could be owing to experiences of minority stress.^21,22^ The minority stress model asserts that individuals who identify with and belong to minority groups (eg, gender minorities, such as transgender people) are likely to experience greater levels of stress due to several factors, including interpersonal, social, and structural forms of stigma and discrimination. Greater levels of stress are likely to place individuals in minority groups at elevated rates of coping behaviors, including substance use and smoking.^23,24^ Minority stress is particularly applicable to transgender adolescent girls and young women who reported having other sexual orientation types (eg, pansexual, asexual), in part because they may not fully belong to or identify with other more common sexual orientations (eg, heterosexual, gay, lesbian) that may have community-centered support that can buffer against minority stress.^25^ As such, they may experience anxiety related to identifying with other sexual orientation types, which can influence stress levels as well as increase behaviors related to substance use, including cigarette smoking and nonmedical prescription opioid use. Future research should further examine this postulation, particularly regarding the role of social support, which has been posited to help alleviate stress and reduce substance use in the context of the minority stress model.^25^

Social participation and cohesion theories^26,27,28^ may also help explain why transgender adolescent girls and young women with other sexual orientation types reported higher nonmedical prescription opioid use. These theories highlight the role of community engagement to increase mutual aid, trust, and solidarity among group or community members and reduce negative health behaviors, like substance use,^29,30^ which could also help explain why differences were not observed among gay, lesbian, or bisexual transgender adolescent girls and young women. Given that gay, lesbian, and bisexual groups in the United States have historically and relatively been more formalized and mobilized as communities or networks^31,32^ compared with those who belong to other kinds of sexual orientations, it is likely that such social cohesiveness and participation among gay and lesbian transgender adolescent girls and young women may be associated with lower nonmedical prescription opioid use. Future research should also further investigate individuals whose sexual orientation is different from heterosexual, gay, lesbian, or bisexual.

### Strengths and Limitations

This study has several strengths. One major strength was the characterization of risk factors of lifetime nonmedical prescription opioid use specifically among transgender adolescent girls and young women. Studies among transgender populations have often considered substance use as a factor associated with HIV risks, which limits our understanding about disparities related to substance use and factors that are associated with substance use among this population.^19^ In contrast to this predominant approach in transgender research, the analysis approach of this study specifically explored risk factors associated with lifetime nonmedical prescription opioid use.

Our analysis also has several limitations. First, we used a non–probability sampling approach to recruit participants for the study, which may have resulted in selection bias. Second, the sample was drawn from a randomized clinical trial that had strict eligibility criteria, which might make the results not generalizable to the population of interest. Third, the original study was not powered to detect possible differences in nonmedical prescription opioid use; thus, it may be underpowered to detect meaningful differences in some outcomes. Fourth, the cross-sectional nature of the study precludes us from any conclusions regarding causality. The sample represents a subset of transgender people and is therefore not representative of the entire transgender community, many of whom have gender expansive or gender nonconforming identities. Additionally, the self-reported nature of the data collection may bias directionality of the results such that participants may have felt stigmatized to disclose or reveal their opioid use and other personal behaviors and experiences. Future research should include biomarkers to determine actual prevalence.

## Conclusions

This cross-sectional study provides insights into the prevalence of lifetime nonmedical prescription opioid use among transgender adolescent girls and young women living in 2 metropolitan US cities. Evidence-based services for substance use that specifically target opioid use disorder can and should be comprehensively tailored to transgender populations, particularly transgender adolescent girls and young women. Integration of such services with identity-affirming mental health services could also be valuable to reaching and targeting this population. The findings of this study may serve as a warranted call-to-action for public health surveillance studies to specifically examine trends of substance use, particularly opioid use disorder, among transgender populations.
